# Prevalence and genotype distribution of human papillomavirus in Sulaymaniyah, Kurdistan Region, Iraq

**DOI:** 10.3389/fcimb.2026.1830236

**Published:** 2026-06-10

**Authors:** Sirwan Sleman

**Affiliations:** College of Veterinary Medicine, University of Sulaimani, Sulaymaniyah, Iraq

**Keywords:** high-risk HPV cervical cancer, HPV genotyping, HPV multiplex qPCR genotyping, HPV prevalence, human papillomavirus infection

## Abstract

Human papillomavirus (HPV) is a small, non-enveloped double-stranded DNA virus belonging to the family *Papillomaviridae*, with a strong tropism for epithelial tissues and well-established oncogenic potential. The epidemiological data of HPV are limited in the Kurdistan Region of Iraq. In this cross-sectional study, a total of 1350 clinical samples (primarily cervical swabs) were collected from individuals attending private diagnostic laboratories in Sulaymaniyah between November 2022 and June 2025. Samples were preserved in viral transport medium and stored at 2–8 °C prior to DNA extraction using a silica column-based nucleic acid purification method. HPV DNA detection and genotyping were performed using the Bosphore HPV Genotyping High Risk Kit v1 (Anatolia Geneworks, Istanbul, Turkey), a multiplex real-time PCR-based system. The results revealed an overall HPV prevalence of 19.2% (261/1350), with HPV-51 being the most common circulating genotype (32.15%), followed by HPV-16 (30.66%), HPV-31 (22.63%), HPV-52 (15.33%), HPV-66 (14.94%), HPV-35 (11.86%), HPV-45 (11.49%) and HPV-56 (11.11%). Multiple genotype infections were observed in more than half of the infected cases. These findings demonstrate a moderate prevalence of high-risk HPV in Sulaymaniyah, with an unusual predominance of HPV-51, suggesting a distinct regional genotype pattern. The absence of vaccination and high rate of co-infections highlight significant public health concerns, emphasising the urgent need for implementing HPV vaccination programs and strengthening screening strategies in the region.

## Introduction

Human papillomavirus (HPV) infection is among the most prevalent sexually transmitted viral infections worldwide and the leading cause of cervical, anogenital, and oropharyngeal cancers (Doorbar et al., 2020; Yousefi et al., 2020). HPV is a small, non-enveloped, double-stranded DNA virus belonging to the family Papillomaviridae, with more than 200 identified genotypes classified into low-risk and high-risk types based on oncogenic potential (Doorbar et al., 2020). High-risk HPV types, including HPV-16, -18, -31, and -51, are strongly associated with malignant transformation through the expression of viral oncoproteins (E6 and E7), which interfere with tumour suppressor pathways such as p53 and retinoblastoma protein (pRb) (Doorbar et al., 2020; Yousefi et al., 2020).

HPV is primarily transmitted through sexual contact, including vaginal, anal, and oral routes, although vertical (perinatal) transmission has also been reported (Yousefi et al., 2020). Persistent infection with high-risk HPV types is the principal cause of cervical cancer, accounting for approximately 99% of cases worldwide (Bruni et al., 2023; World Health Organization (WHO), 2024). Despite the availability of effective vaccines, HPV remains a major global public health challenge, particularly in low- and middle-income countries where vaccination programs are not fully implemented (de Martel et al., 2017; Bruni et al., 2023).

Globally, HPV prevalence varies widely depending on geographic region and population characteristics, ranging from 10% to over 20%, with higher rates reported in developing regions (Forman et al., 2012; Bruni et al., 2023). Large-scale epidemiological studies have demonstrated significant variation in genotype distribution across populations, with HPV-16 and HPV-18 being the most dominant types worldwide, although other genotypes such as HPV-31, HPV-52, and HPV-51 are increasingly reported in certain regions (Han et al., 2025; Ke et al., 2025).

In the Middle East, HPV prevalence and genotype distribution exhibit notable variability. Studies from Iran have reported prevalence rates as high as 38.7%, with diverse genotype patterns (Gorur et al., 2022). Similarly, research from Turkey and Saudi Arabia indicates the circulation of multiple high-risk HPV types, including HPV-16, -18, -31, and -51 (Jihad et al., 2020; Alobaid et al., 2022).

In Iraq, available data remain limited. Previous studies conducted in Baghdad and other regions have reported HPV prevalence rates ranging from 16.7% to 19%, with HPV-16 and HPV-18 as the dominant genotypes (Burd, 2016; Marhash et al., 2025; Tahir et al., 2025). However, comprehensive molecular epidemiological data from the Kurdistan Region, particularly Sulaymaniyah, are scarce.

Understanding regional HPV genotype distribution is essential for guiding vaccination strategies and cervical cancer prevention programs. Given that currently available vaccines (e.g., the nonavalent vaccine) target specific HPV genotypes, local epidemiological data are critical to evaluate their effectiveness in different populations (Bruni et al., 2023).

Therefore, this study aimed to determine the prevalence and genotype distribution of high-risk HPV among individuals tested in Sulaymaniyah between November 2022 and June 2025 using a validated multiplex real-time PCR-based genotyping system. These findings provide important baseline data for HPV surveillance and public health planning in the region.

## Materials and methods

### Study design and setting

A cross-sectional molecular epidemiological study was conducted using routinely collected HPV PCR test results from private diagnostic laboratories in Sulaymaniyah, Kurdistan Region of Iraq. The current study duration was between November 2022 and June 2025, with all available high-risk HPV genotyping profiles retrieved and analysed. Laboratory methods adhered to validated clinical molecular diagnostics protocols to permit accurate diagnosis and genotyping of high-risk HPV types.

### Study population and sample collection

A dataset of 1350 individuals who had HPV PCR testing for routine clinical evaluation was provided. Cervical swabs were harvested by sterile Dacron or nylon flocked swabs and placed instantaneously in viral transport medium post‐samples. All samples were labelled with anonymous laboratory identifiers and taken on the day of collection or kept at 2-8 °C for 24 h before DNA extraction. Demographic samples (age, sex, blood group) were also recorded.

### Inclusion and exclusion criteria

Inclusion criteria included individuals who underwent HPV PCR testing during the study period with complete demographic and laboratory data. Exclusion criteria included incomplete records, degraded samples, or invalid PCR results due to failed internal controls.

### Data collection

Data were retrospectively collected from laboratory records and included demographic variables (age, sex), sample type, HPV test results, and genotype profiles. All data were anonymised before analysis.

### Ethical considerations

Ethical approval for this study was obtained from the Institutional Review Board (IRB) of the National Institute of Technology (Protocol No. NIT-IRB-NUR-2022-017). Patient confidentiality was strictly maintained, and all data were anonymised.

### DNA extraction

Genomic DNA was extracted by using a commercially available silica-based nucleic acid extraction kit (Qiagen, Germany) according to the manufacturer’s protocol. Briefly, clinical specimens were lysed with chaotropic reagents that ruptured epithelial cells and released HPV DNA. Lysates were then transferred to spin columns, where nucleic acids selectively bound to the silica matrix under high-salt conditions. DNA was eluted in nuclease-free buffer after multiple wash steps to remove proteins, inhibitors, and debris and stored at −20 °C until PCR amplification. Extraction accuracy and PCR optimisation were evaluated with internal control amplification in all the reaction fractions.

### HPV detection and genotyping assay

**Figure 1:**
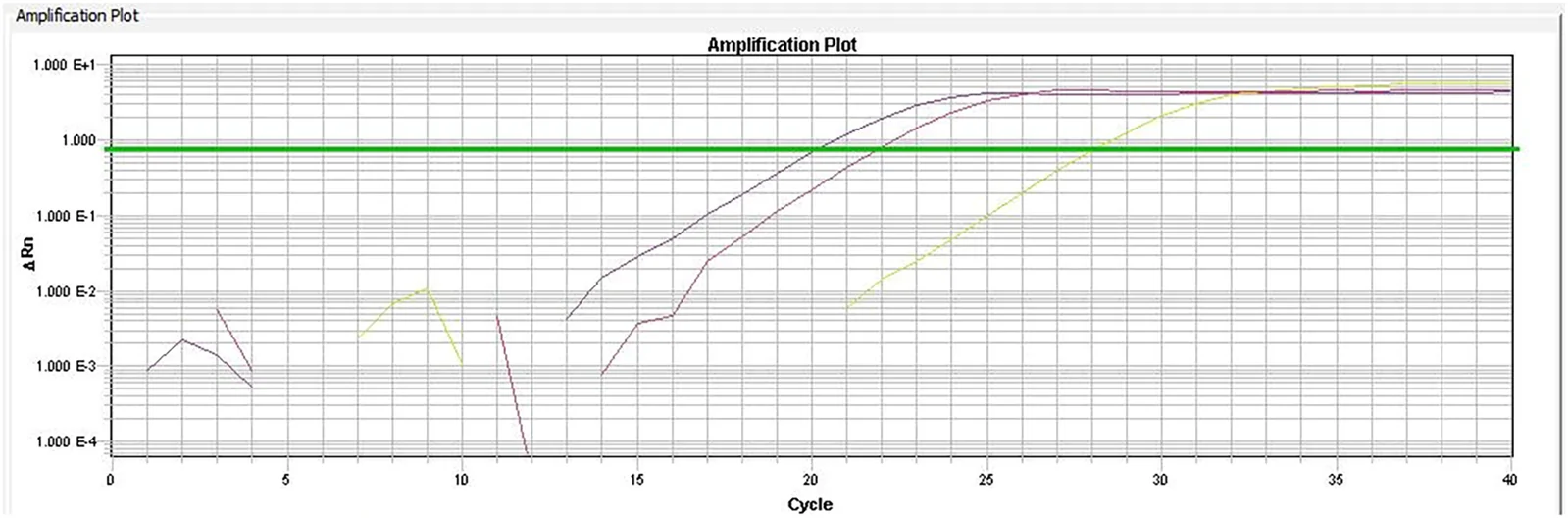
PCR amplification plot. The plot shows single representative qPCR amplification curves with Ct values at ~22 for the positive control (Red), ~20 for HPV-51 (purple), and ~28 for HPV-66 (yellow). The presence of two distinct target curves indicates co-infection with HPV-51 and HPV-66, with HPV-51 amplifying earlier, consistent with a higher viral load.

High-risk HPV detection and genotyping were carried out using the Bosphore HPV Genotyping High Risk Kit v1 (Anatolia Geneworks, Istanbul, Turkey) (**Supplementary Table 1**). This commercial multiplex real-time PCR assay amplifies the L1 portion of the HPV genome and detects major high-risk HPV genotypes, namely HPV-16, 18, 31, 33, 35, 39, 45, 51, 52, 56, 58, 59, 66 and 68. Genotype-specific primer–probe complexes labelled with fluorophores (FAM, HEX, ROX, Cy5) provide the ability to recognise several genotypes simultaneously in a single tube, as demonstrated by the kit. Each reaction contained an internal amplification control that was responsible for verifying both extraction quality and ruling out PCR inhibition. Fluorogenic hydrolysis probes (TaqMan chemistry) are employed to gain high analytical specificity and high sensitivity for clinical diagnosis (**Figure 1**).

### PCR reaction setup and thermal cycling

PCR reactions were prepared in a designated pre-amplification area to reduce contamination. Each 25 µL reaction mix consisted of the master mix with hot-start Taq DNA polymerase, dNTPs and optimised MgCl_2_ concentration, genotype-specific primers and fluorescent probes, internal control template and primers, and 5–10 µL of purified DNA. The tubes were sealed and vortexed briefly before centrifugation and loaded onto a real-time PCR instrument compatible with detection channels needed for the assay. Cycling conditions were according to the manufacturer’s recommended thermal program, with initial denaturation, 35–40 cycles of denaturation, annealing, and extension, and fluorescence acquisition at each cycle. Amplification curves were automatically recorded for each fluorophore channel, allowing for discrimination of individual HPV genotypes and detection of mixed infections.

### Quality control measures

For each PCR reaction, a positive control DNA was included for assay verification, a no-template negative control was added to test for possible contamination, and an internal control amplification was performed for each sample to evaluate extraction efficiency and check for PCR inhibition.

### Data interpretation

Data for amplification were processed by use of the instrument’s proprietary PCR software. A sample was considered HPV-positive when a genotype-specific fluorescence signal crossed the threshold line within the acceptable Ct range. Samples showing no HPV amplification but a valid internal control were classified as HPV-negative. Multiple concurrent fluorescence signals interpreted as co-infections. Genotype results were exported into an anonymised dataset and compiled for epidemiological analysis.

### Statistical analysis

All data were descriptively analysed with Microsoft Excel and statistical software. Summary statistics were HPV prevalence, genotype frequencies, age distribution, sex distribution, and single infections vs. multiple infections. Continuous variables (e.g., age) were summarised in mean/median/range and categorical variables (e.g., genotype presence) as counts and percentages.

Statistical analysis was performed using IBM SPSS Statistics (version 31, IBM Corp., Armonk, NY, USA). Chi-square tests were used to assess associations between categorical variables, and p-values <0.05 were considered statistically significant.

## Results

### Study population characteristics

Between November 2022 and June 2025, 1350 patients underwent HPV PCR tests. With 1291 female participants (96.6%) and only 59 males (4.4%) in the dataset. The mean age of the study population was 34.5 years (range: 1–70 years), and the period of highest testing frequency was in females aged 25–45 years. All participants were unvaccinated for HPV (**Table 1**).

**Table 1:** Demographic characteristics of the study population (N = 1350).

Characteristic	Category	Estimated frequency (n)	Percentage (%)
Sex	Female	1291	95.6
	Male	59	4.4
Age group (years)	25–45	1002	74.2
	<25 or >45	348	25.8
Blood group	A±	176	13.0
	B±	127	9.4
	AB±	23	1.7
	O±	1034	76.6

### HPV prevalence

Among those 1350 samples analysed, 261 were positive for a high-risk HPV genotype, totalling an overall prevalence of 19.2%. The remaining 1089 samples (80.8%) were negative. All HPV contaminated and positive samples were obtained from females, with no cases from males of HPV origin. The majority of patients with HPV infection were females, aged 25–45 years, who presented the highest burden, accounting for 74.2% of all positive HPV cases (**Table 2**).

**Table 2:** Overall HPV prevalence.

Variable	Estimated frequency (n)	Percentage (%)
Total tested individuals	1350	100
HPV-positive cases	261	19.2
HPV-negative cases	1089	80.8

### Genotype distribution

**Figure 2:**
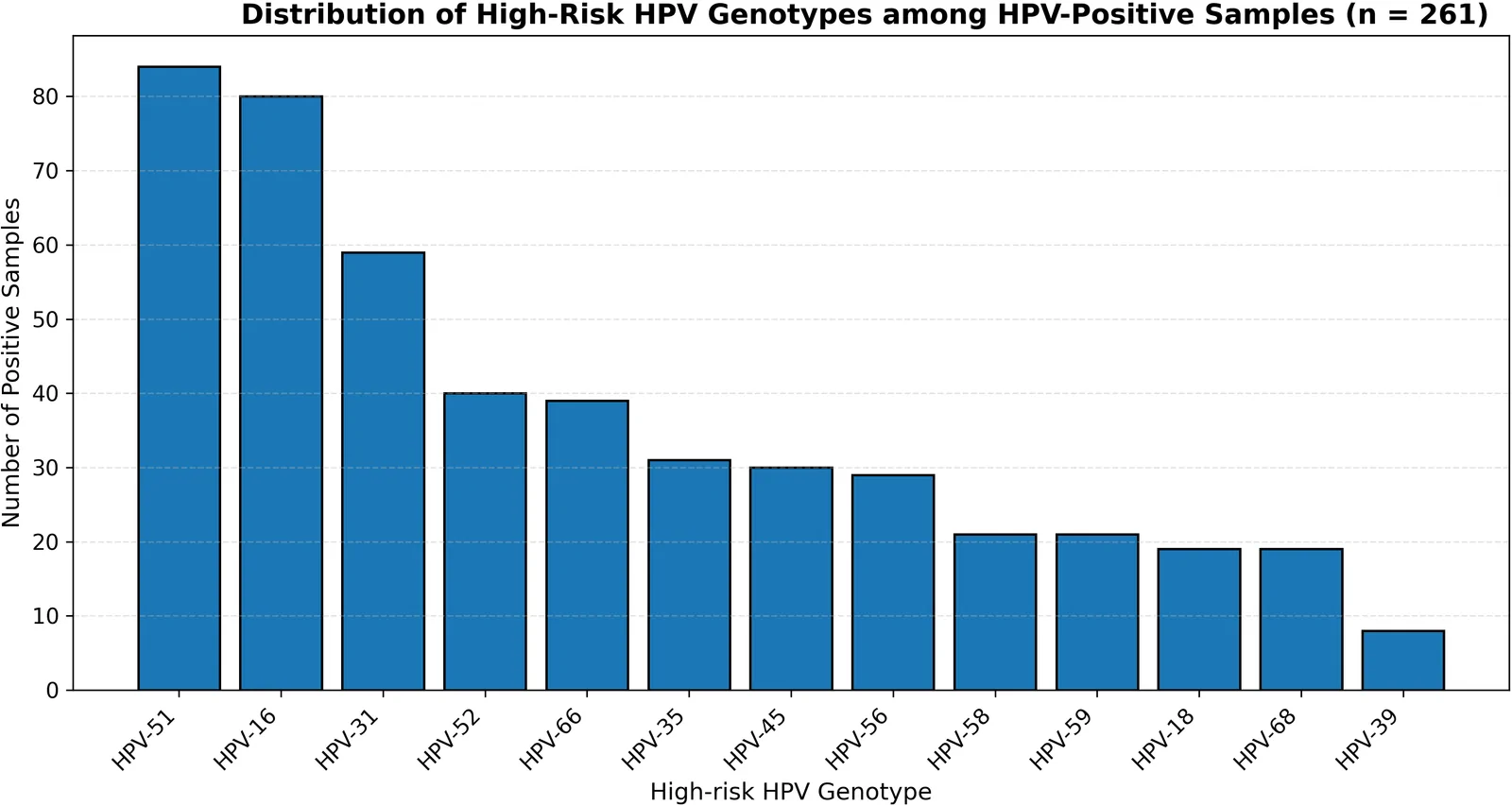
Distribution of High-Risk HPV Genotypes among HPV-Positive Samples (n = 261).

Out of all 261 positive cases, HPV-51 (32.15%, n=84), high-risk HPV genotype was most often detected, followed by HPV-16 (30.66%, n=80), HPV-31 (22.63%, n=59), HPV-52 (15.33%, n=40), and HPV-66 (14.94%, n=39) being the other highly frequent genotypes detected. Rare genotypes were HPV-35, HPV-45, HPV-56, HPV-58, HPV-59 HPV-18, HPV-68, and HPV-39 (**Table 3**). Co-infections, defined as ≥2 genotypes, were detected in more than half of positive samples (53.62%), and the most common co-infected genotypes were HPV-16, 51, 31, 52, and 66. No HPV-33 were detected in this population (**Table 4**; **Figure 2**).

**Table 3:** High-risk HPV genotype distribution among positive samples (n = 261).

Genotype	Estimated frequency (n)	Percentage (%)
HPV-51	84	32.15
HPV-16	80	30.66
HPV-31	59	22.63
HPV-52	40	15.33
HPV-66	39	14.94
HPV-35	31	11.86
HPV-45	30	11.49
HPV-56	29	11.11
HPV-58	21	8.03
HPV-59	21	8.03
HPV-18	19	7.28
HPV-68	19	7.28
HPV-39	8	3.07

Percentages exceed 100% due to multiple-genotype infections.

**Table 4:** Prevalence of single and multiple HPV infections.

Pattern	Numbers	Percentage (%)
Single-genotype infection	121	46.38
Multiple-genotype infection	140	53.62

The predominance of HPV-51 (32.15%) over HPV-16 represents a notable deviation from global trends, where HPV-16 is typically the most prevalent genotype. This finding may reflect regional epidemiological variation or population-specific viral circulation patterns and warrants further large-scale investigation.

Risk-based grouping of genotypes indicated that the majority of detected types belonged to high-risk oncogenic categories, reinforcing the clinical significance of these findings.

### Age distribution of HPV-positive cases

**Figure 3:**
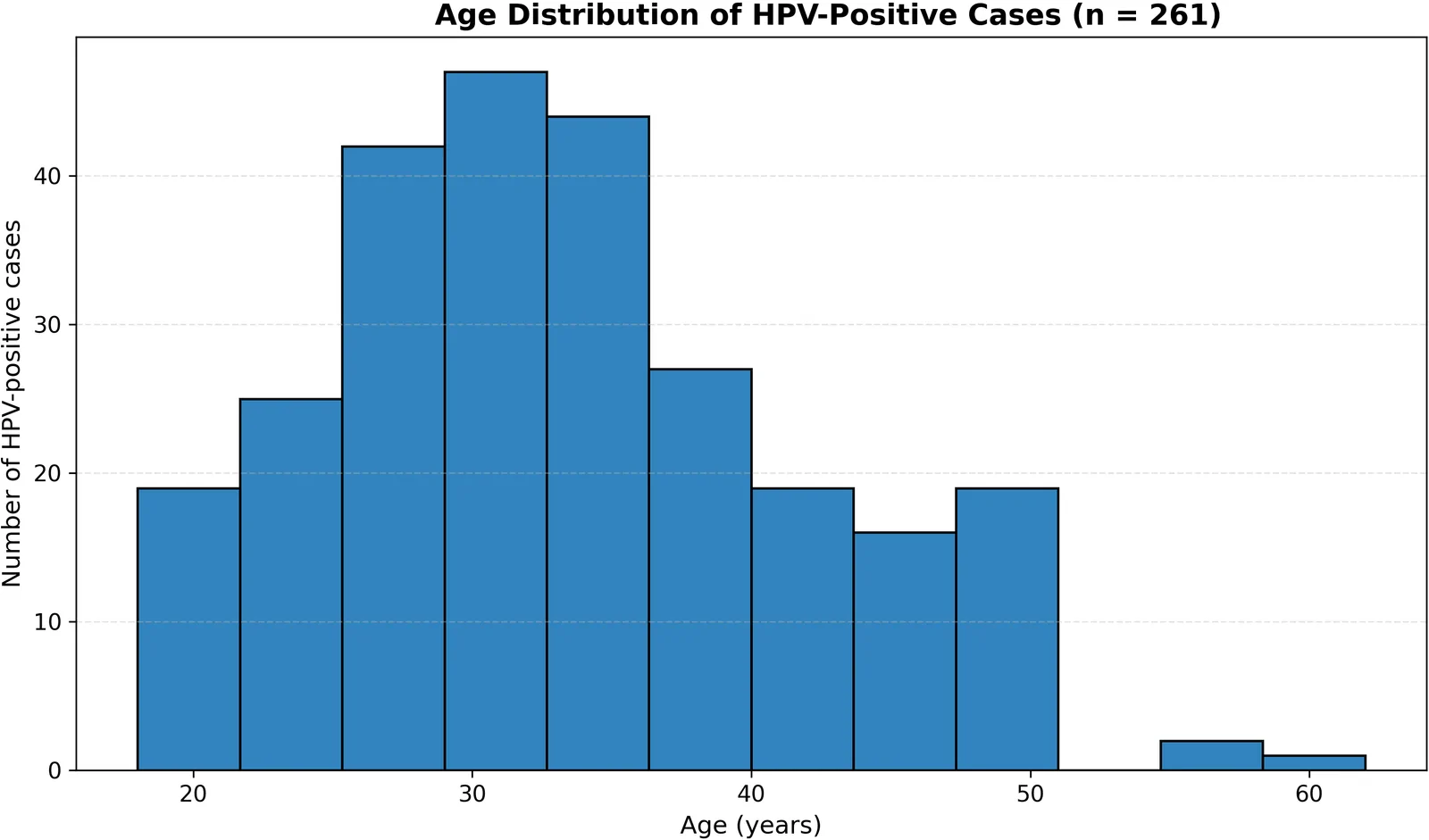
Age Distribution of HPV-Positive Cases (n = 261).

HPV infection was most prevalent among those aged 20 to 40 years old, and the average age within the positive group was 33.8 years (**Figure 3**). There were only two infections in individuals > 50 years, and only one positive case in a 1-year-old child, pointing to perinatal transmission of infection or a false positive reaction that required further clinical evaluation.

The overall findings suggest the prevalence of HPV in Sulaymaniyah is moderate, with unusually high predominance of HPV-51 compared with global patterns. The findings underscore the lack of HPV vaccination in the population and the need for targeted public health interventions.

## Discussion

This study presents the first comprehensive molecular epidemiological analysis of high-risk HPV prevalence and genotype distribution in Sulaymaniyah, Iraq. The overall HPV prevalence of 19.2% observed in this study is consistent with previously reported rates from Iraq, which range between 16.7% and 19% (Burd, 2016; Marhash et al., 2025; Tahir et al., 2025). Comparable prevalence rates have also been reported in neighbouring Middle Eastern countries, such as Turkey (approximately 23%) and Saudi Arabia (14.6%), indicating a moderate regional burden of HPV infection (Jihad et al., 2020; Alobaid et al., 2022).

One of the most notable findings of this study is the predominance of HPV-51 (32.15%), exceeding the prevalence of HPV-16. Globally, HPV-16 and HPV-18 are typically the most prevalent genotypes and account for the majority of cervical cancer cases (Doorbar et al., 2020; Bruni et al., 2023). However, emerging evidence suggests that genotype distribution varies geographically, with increasing detection of HPV-31, HPV-52, and HPV-51 in certain populations (Tayyar et al., 2019; Han et al., 2025; Ke et al., 2025). Large-scale studies involving millions of participants have demonstrated significant regional variation in HPV genotype patterns, supporting the findings of this study (Han et al., 2025). The unusually high prevalence of HPV-51 in Sulaymaniyah may therefore reflect a distinct regional epidemiological pattern, highlighting the importance of localised surveillance.

The complete absence of HPV vaccination among study participants is another critical finding. This observation is consistent with national data indicating that Iraq has not yet implemented a widespread HPV immunisation program. The lack of vaccination allows continued circulation of high-risk HPV genotypes, including vaccine-preventable types such as HPV-16 and HPV-18 (Bruni et al., 2023; World Health Organization (WHO), 2024). The presence of multiple vaccine-covered genotypes (HPV-16, 18, 31, 45, 52, and 58) in this study underscores the potential public health benefit of introducing the nonavalent HPV vaccine in the region.

Co-infections were observed in more than half of HPV-positive cases, which is higher than some previously reported rates. Multiple HPV infections have been associated with increased risk of persistent infection and progression to cervical neoplasia (Yousefi et al., 2020). This finding emphasises the importance of comprehensive genotyping rather than simple HPV detection, as co-infections may influence disease progression and clinical outcomes.

The age distribution of HPV-positive individuals in this study showed the highest prevalence among women aged 25–40 years, which aligns with global epidemiological trends (Forman et al., 2012). This age group represents the peak of sexual activity and exposure risk, as reported in multiple international studies (Bruni et al., 2023; Han et al., 2025). The detection of HPV in a very young child, although rare, raises the possibility of perinatal transmission or laboratory-related factors, which should be interpreted cautiously.

Comparison with global data further highlights the importance of regional differences. Large multicentre and population-based studies from China and other countries have demonstrated dynamic changes in HPV prevalence and genotype distribution over time, influenced by factors such as vaccination, screening programs, and public health interventions (Han et al., 2025; Ke et al., 2025; Liu et al., 2025). These findings reinforce the need for continuous epidemiological monitoring in Iraq.

Despite the strengths of this study, including a relatively large sample size and the use of a validated multiplex real-time PCR assay, several limitations should be acknowledged. The study population consisted of individuals undergoing clinical testing and may not represent the general population. Additionally, the lack of cytological or histopathological correlation limits the ability to assess the clinical significance of specific genotypes.

Overall, this study provides important baseline data on HPV epidemiology in Sulaymaniyah and highlights the urgent need for implementing HPV vaccination programs, expanding cervical cancer screening, and conducting further large-scale studies to better understand genotype distribution and associated risks in the Iraqi population.

## Conclusion

This report shows a 19.2% prevalence of high-risk HPV infection among individuals tested in Sulaymaniyah, with the highest infection rates observed in women aged 25–40 years. HPV-51 emerged as the most dominant genotype (32.15%), exceeding the global predominance of HPV-16/18 and indicating a distinct regional epidemiological pattern.

Other more common high-risk HPV genotypes recorded were HPV-16, HPV-31, HPV-52, and HPV-66. Multiple high-risk HPV co-infections occurred in more than half of positive cases, underscoring the importance of comprehensive genotyping rather than single-target HPV testing. None of the study participants was vaccinated, highlighting an urgent public health need for implementing HPV vaccination programs in Iraq.

In Iraq, the failure to vaccinate against HPV in the analysed group exposes a significant gap in public health. The implementation of HPV vaccination, expansion of cervical screening facilities, and an increase in the implementation of large multi-centre epidemiological studies are necessary to reduce cervical cancer and HPV-related disease burden in the region. The establishment of these findings represents substantial baseline evidence for regional public health programs, vaccine policy, and future research of HPV epidemiology in the region.

This study provides important baseline epidemiological data and highlights the need for national HPV surveillance programs, implementation of vaccination strategies, and integration of genotype-specific risk assessment into cervical cancer prevention frameworks in Iraq.

## Data Availability

The original contributions presented in the study are included in the article/**Supplementary Material**. Further inquiries can be directed to the corresponding author.
