# Correction: Prevalence and genotype distribution of human papillomavirus in Sulaymaniyah, Kurdistan Region, Iraq

**DOI:** 10.3389/fcimb.2026.1959601

**Published:** 2026-08-10

**Authors:** Sirwan Sleman

**Affiliations:** College of Veterinary Medicine, University of Sulaimani, Sulaymaniyah, Iraq

**Keywords:** high-risk HPV cervical cancer, HPV genotyping, HPV multiplex qPCR genotyping, HPV prevalence, human papillomavirus infection

Affiliation “Nursing Department, National Institute of Technology, Sulaymaniyah, Iraq” was erroneously included in the paper. This affiliation has now been removed for author “Sirwan Sleman”.

The original version of this article has been updated.

