# Supplementary material for: Prevalence and genotype distribution of human papillomavirus in Sulaymaniyah, Kurdistan Region, Iraq
Source: Front Cell Infect Microbiol. 2026 Jun 10;16:1830236. doi: 10.3389/fcimb.2026.1830236 (PMC13291117; doi:10.3389/fcimb.2026.1830236)
Supplement: Supplementary file 1 [file Table1.docx]

**Supplementary Materials**

**Prevalence and Genotype Distribution of Human Papillomavirus (HPV) in Sulaymaniyah, Kurdistan Region of Iraq.**

**Authors:**

Sirwan Sleman ^1,2,*^

**Affiliations:**

¹ College of Veterinary Medicine, University of Sulaimani, Sulaymaniyah, Iraq.

^2^ Nursing Department, National Institute of Technology, Sulaymaniyah, Iraq.

***Corresponding Author:** Assist. Prof. Dr Sirwan Sleman

**Email**: [sirwan.sleman@univsul.edu.iq]

**Phone**: [+9647721525583]

**ORCID** IDs: [Sirwan Sleman (0000-0001-8589-0345) - ORCID](https://orcid.org/0000-0001-8589-0345)

**Running Title:** HPV Prevalence and Genotype Distribution

**Tables S1. Materials and Reagents (Bosphore kit).**

| Category | Material / Reagent | Manufacturer / Catalog Details | Purpose |
| --- | --- | --- | --- |
| Sample Collection | Cervical swab collection kit | Sterile single-use | Sample acquisition |
| DNA Extraction | Silica-membrane DNA extraction kit | Commercial column system | HPV DNA purification |
| PCR Kit | *Bosphore HPV Genotyping High Risk Kit v1* | Anatolia Geneworks, Turkey | Multiplex HR-HPV detection and genotyping |
| PCR Components | Master mix with dNTPs, Hot-Start Taq polymerase | Included in the Bosphore kit | DNA amplification |
|  | Genotype-specific primers & fluorescent probes | Included in the Bosphore kit | HPV genotype identification |
| Controls | Internal amplification control | Included in kit | PCR inhibition detection |
|  | Positive and negative controls | Provided by the kit | Assay validation |
| Equipment | Real-time PCR thermocycler (FAM, HEX, ROX, Cy5 channels) | Compatible instrument | Multiplex fluorescence detection |
| Consumables | Nuclease-free water | Molecular grade | Reaction preparation |
|  | PCR tubes/plates, filter tips | Standard molecular supplies | Contamination prevention |
